# Supplementary material for: Neo-epidemiological machine learning based method for COVID-19 related estimations
Source: PLoS One. 2023 Mar 24;18(3):e0263991. doi: 10.1371/journal.pone.0263991 (PMC10038265; doi:10.1371/journal.pone.0263991)
Supplement: Attachment — Submitted filename: response to reviewer.pdf [file pone.0263991.s005.pdf]

Response to Reviewer

<table>
<tr><th></th><th>Question</th><th>Response</th></tr>
<tr><td>1</td><td>Authors need to highlight the research gap and highlight the contribution(s) of the research work in the manuscript to shows the novelty of the research.</td><td>On page 5 before listing the contributions of the present study the following items were added:
According to the above said issues and studying similar content, the general gaps existing in the body of literature can be listed as below:
1- All models estimating the basic production number need reliable and precise information.
2- The previous models estimating the basic production number have worked on narrow data, and do not cover large data and those with noise.
3- Parameters of the previous models estimating the basic production number are not flexible and do not work with the same data.
4- Discovery process of epidemiological knowledge in the previous models estimating the basic production number is not possible without the mastery and knowledge of an expert epidemiologist.</td></tr>
<tr><td>2</td><td>I will suggest author to perform a sensitivity analysis to make the results to be more convincing.</td><td>Due to the fact that extracting results with large data requires time processing based on artificial intelligence systems, so adding another evaluation index for the present study requires a lot of time, but in any case the following comparison table was added to the results section:
Table 17 shows the superiority of the present study's method in terms of processing with accurate data, the possibility of processing with low data volume and noisy data, and the process of epidemiological knowledge discovery. The present method not only does not require accurate data and has the ability to go through the process of predicting epidemiological processes with accessible data, but also the process of discovering epidemiological knowledge in the present study does not require expert knowledge and with any level of knowledge the epidemiological knowledge can be understood.

Table 17: The superiority of the present study's method compared to the other methods
<table>
<tr><th>Parameter</th><th>Present study</th><th>Previous studies</th></tr>
<tr><td>Precise and reliable data</td><td>Does not needs precise and reliable data</td><td>Needs precise and reliable data</td></tr>
<tr><td>Processing possibility with low and noisy data</td><td>Processing low and noisy data is possible</td><td>Processing low and noisy data is not possible</td></tr>
<tr><td>The discovery process of epidemiological knowledge</td><td>Discovery process of epidemiological knowledge does not need an expert epidemiologist</td><td>Discovery process of epidemiological knowledge needs an expert epidemiologist</td></tr>
</table>
</td></tr>
</table>

3	More critical discussion is needed on the results and discussion. The current one is not sufficient. E.g. why the small improvement is significant?	The following content was added to the discussion section:<br>The importance of the improvements that have taken place in the results lies in the fact that these results are obtained with data that the accuracy of all this data is not verifiable and in other words we do not encounter reliable data. Therefore, slight improvements in the results of the present study with data obtained in the early COVID-19 pandemic period promise that when reliable data are not available, we can rely on the learning method of the present study, which over time due to learning, the system's answers are more accurately and complete. On the other hand, the improved obtained answers are not necessarily obtained with the help of an expert epidemiologist, and with any level of epidemiological knowledge, knowledge can be extracted. Another problem that adds to the value of the responses obtained is that by processing large volumes of data that are definitely noisy, a response worthy of attention with little improvement over previous methods is obtained.
4	More recent references can be added to the manuscript.	The following references have been added to the article:<br>38. Soudeh Ghafouri-Fard and Hossein Mohammad-Rahimi and Parisa Motie and Mohammad A.S. Minabi and Mohammad Taheri and Saeedeh Nateghinia. Application of machine learning in the prediction of COVID-19 daily new cases: A scoping review *H*eliyon,7:e08143.2021. doi :10.1016/j.heliyon.2021.e08143.<br>39. Dandekar, Raj and Barbastathis, George. Quantifying the effect of quarantine control in Covid-19 infectious spread using machine learning. *C*old Spring Harbor Laboratory Press. elocation-id: 2020.04.03.20052084. 2020. doi :10.1101/2020.04.03.20052084.<br>40. Sujath, R. and Chatterjee, Jyotir Moy and Hassanien, Aboul Ella. A machine learning forecasting model for COVID-19 pandemic in India. *S*tochastic Environmental Research and Risk Assessment. .959-972.2020.
